# *HBB*-deficient *Macaca fascicularis* monkey presents with human β-thalassemia

**DOI:** 10.1007/s13238-019-0627-y

**Published:** 2019-05-20

**Authors:** Yan Huang, Chenhui Ding, Puping Liang, Duanduan Li, Yu Tang, Wei Meng, Hongwei Sun, Hongyu Lu, Yu Chen, Xueying Chen, Qunshan Huang, Jianpei Fang, Canquan Zhou, Shihua Yang, Junjiu Huang

**Affiliations:** 1 College of Veterinary Medicine & Key Laboratory of Comprehensive Prevention and Control for Severe Clinical Animal Diseases of Guangdong Province, South China Agricultural University, Guangzhou, 510642 China; 2 Key Laboratory of Reproductive Medicine of Guangdong Province, The First Affiliated Hospital and School of Life Sciences, Sun Yat-sen University, Guangzhou, 510275 China; 3 MOE Key Laboratory of Gene Function and Regulation, State Key Laboratory of Biocontrol, School of Life Sciences, SunYat-sen University, Guangzhou, 510275 China; 4 Department of Pediatrics, Second Affiliated Hospital, Sun Yat-sen University, Guangzhou, 510120 China

**Dear Editor,**

β-Thalassemia is a common severe genetic disease caused by mutations in *HBB* and affects approximately 1.5% of the global population (Origa, 2017). In southern China, the carrier rate of β-thalassemia is as high as 6.43%, creating a high socio-economic burden (Xiong et al., 2010). In adult humans, there are three types of hemoglobin: HbA1 (~97%), HbA2 (~2%) and HbF (~1%). HbA1 (α_2_β_2_) is composed of two α-globin and two β-globin subunits encoded by *HBA* and *HBB*, respectively; HbF (α_2_γ_2_) is made up of two α-globin subunits and two γ-globin subunits encoded by *HBG*. Mutations in the coding region or regulatory region of *HBB* are involved in β-thalassemia pathogenesis. Except for some rare dominant mutations, most *HBB* mutations are recessive (Origa, 2017). Depending on the mutation type, the β-globin level will either be reduced or completely depleted, resulting in α-globin accumulation and precipitation. These α-globin precipitates lead to red blood cell death, resulting in anemia and tissue damage, and even death in β-thalassemia major patients. Blood transfusions can help slow disease progression but lead to iron overload, ultimately resulting in iron toxicity. Bone marrow transfer is the only cure in the clinic and is available only to a small percentage of patients with human leukocyte antigen-matched donors. Recently, gene therapy and gene editing therapy have shown great promise in curing β-thalassemia (Glaser et al., 2015; Thompson et al., 2018). However, no appropriate animal models are available for evaluating the safety and efficacy of such advanced therapeutic strategies *in vivo*. β-thalassemia mice are the sole animal model available for research. However, substantial differences have been reported between the types and expression patterns of human and mouse globins (McColl and Vadolas, 2016). Moreover, mice contain no fetal globin gene equivalent, and homozygous mutations of *HBB* in mouse for early models of β-thalassemia major or Cooley anemia are all embryonic lethal (Huo et al., 2009). Recently, significant phenotype and physiology differences have been reported between SIRT6-null mice and the non-human primate model (Zhang et al., 2018). Thus, an appropriate non-human primate model is needed for human β-thalassemia studies and treatments.

*Macaca fascicularis*, a close relative of human, shares many physiological and developmental similarities with human. Genome edited *M*. *fascicularis* models are invaluable for mimicking human diseases (Phillips et al., 2014). In *M*. *fascicularis*, the composition of the β-globin and β-like globin gene locus is very similar to the human counterparts (Supplementary information, Fig. S1). Moreover, *M*. *fascicularis* expresses HbA and HbF at birth, but only HbA in adults (Scott et al., 1986). Whether *HBB* mutant *M*. *fascicularis* recapitulates human β-thalassemia remained unclear.

In this study, we designed three guide RNAs (gRNAs, G1, G2 and G3) targeting the *M*. *fascicularis HBB* gene locus (Supplementary information, Fig. S2). Three gRNAs and Cas9 mRNAs mixture were injected into the cytoplasm of 97 zygotes of *M*. *fascicularis* to generate *HBB* knockout (KO) monkeys. The injected zygotes were then cultured for 7 days, and 32 zygotes developed to blastocysts. In 10 of the 32 blastocysts, *HBB* loci were amplified by PCR after whole-genome amplification. A T7E1 assay, combined with Sanger sequencing, showed that 80% (8/10) of embryos were cleaved by Cas9 nuclease (Supplementary information, Fig. S3). TA cloning and sequencing revealed different mutant alleles, including insertions and deletions. Five of the 8 embryos were showed complete KO without the wild-type (WT) allele (Supplementary information, Fig. S4). Another 22 frozen blastocysts were thawed and transplanted into 7 surrogates, resulting in the production of one new-born monkey, marked as the founder monkey (Fig. 1A). Ear and blood samples were used to amplify the *HBB* locus in this monkey, producing a band approximately 200 base pairs smaller than that in the WT monkey (Supplementary information, Fig. S5A). To test whether there was a PCR bias towards the smaller band in genotyping, the genomic DNA of the founder monkey was diluted with genomic DNA from WT monkey, and we found that we could still detect the WT band even at 160-fold dilution (Supplementary information, Fig. S5B). Next, Sanger sequencing and sequencing alignment revealed pure 217-base pair deletions at the *HBB* locus in both the ear and blood tissues. Taken together, these data suggested that this founder monkey might be a homozygous KO monkey (Fig. 1A and 1B). Next, we searched for potential off-target sites of these three gRNAs by Cas-OFFinder and the top 10 sites of each gRNA were PCR amplified for Sanger sequencing to investigate off-target effects. None off-target cleavage was found (Supplementary information, Fig. S6). In addition, we also found three *HBD* gene loci that were similar to the target sequence of G1, G2 and G3. Fortunately, we found neither off-target cleavage at these *HBD* loci, nor large deletion or inversion between *HBD* and *HBB* (Supplementary information, Fig. S7).

**Figure 1:**
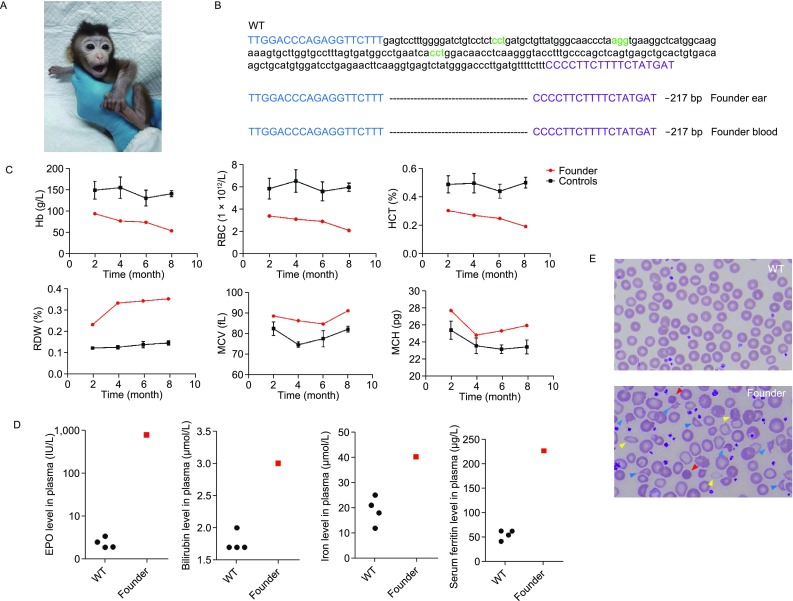

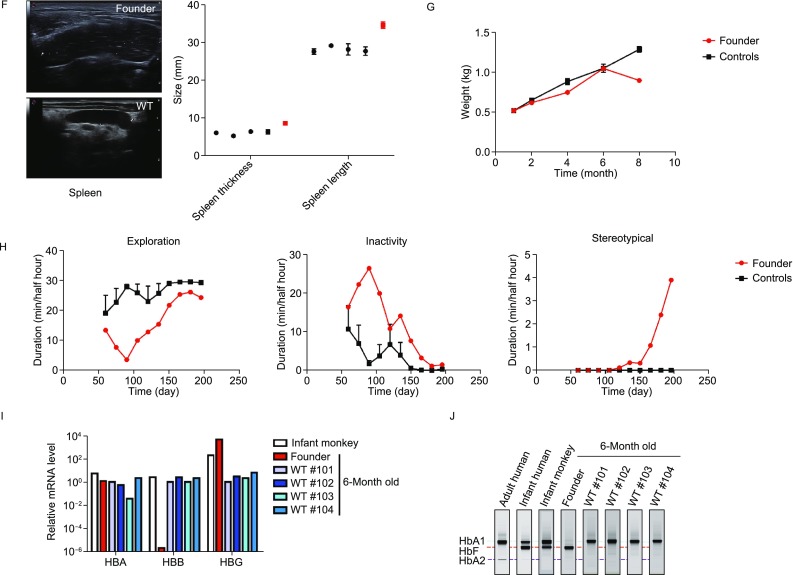
**Generation and identification of** ***HBB*-deficient monkey**. (A) Photo of 60-day-old *HBB*-deficient founder monkey. (B) Sequence alignment of WT monkey and *HBB* KO founder. Deleted base pairs in *HBB* KO founder monkey are in lowercase. Base pairs ahead of the deleted base pairs are in blue uppercase. Base pairs behind the deleted base pairs are in purple uppercase. (C) Routine blood test of the *HBB* KO founder at ages of 2–6 months. (D) Plasma erythropoietin (EPO), bilirubin, iron and serum ferritin analysis. (E) Peripheral blood smear of *HBB* KO founder showed apparent anisocytosis and poikilocytosis. Spherocytes indicated by red triangle. Tear drops indicated by blue triangle. Ovalocytes indicated by yellow triangle. (F) B-ultrasonography spleen of *HBB* KO founder and WT monkeys. (G) The body weight of *HBB* KO founder compared to WT monkeys. (H) Behavioral analysis of *HBB* KO founder and WT monkeys, including active interaction, passive interaction, and stereotypical behaviors. (I) Q-PCR to quantify the mRNA level of *HBA* (*HBA1* and *HBA2*), *HBB* and *HBG* (*HBG1* and *HBG2*). (J) Capillary electrophoresis to quantify hemoglobin level

We next investigated whether the *HBB* KO founder showed the β-thalassemia phenotype. Compared with WT control, the skin of *HBB* mutant founder monkey was much paler, in consistent with anemia phenotype of human patients (Fig. 1A). In addition, blood was collected from age 2 to 8 months for routine blood testing. Similar to human β-thalassemia patients, the value of hemoglobin (Hb), red blood cell (RBC) and hematocrit (HCT) were decreased relative to those of the WT controls (Fig. 1C). Moreover, the value of RBC distribution width (RDW) was elevated compared to wild-type control (Eldibany et al., 1999). But unlike human patients, the values of mean corpuscular volume (MCV) and mean corpuscular hemoglobin (MCH) were increased in this founder monkey (Fig. 1C) (Eldibany et al., 1999). Additionally, the erythropoietin, bilirubin, serum iron, and serum ferritin were increased compared to in the WT monkey, in consistent with those of the human patients (Fig. 1D) (Nienhuis and Nathan, 2012). At the age of 6 months, the blood smear from the *HBB* KO founder monkey revealed obviously abnormal red blood cells, indicating classical erythrocytopoiesis defect in β-thalassemia (Fig. 1E). β-Thalassemia will lead to enlarged spleen and liver, so we observed the spleen and liver by B-ultrasonography. And we found significant splenomegaly, but not hepatomegaly (Fig. 1F and Supplementary information, Fig. S8). Consistent with the anemia phenotypes, the body weight of the *HBB* KO founder was reduced at 8 months of age compared to the WT controls (Fig. 1G). We also recorded and analyzed the daily activities of the monkeys by video to assess their behaviors and found that the *HBB* KO founder was more inactive than WT monkeys (Fig. 1H). Taken together, these phenotypes of this *HBB* KO founder resembles those of human β-thalassemia patients, indicating that *HBB* mutant *M*. *fascicularis* is a good model to study β-thalassemia.

We next evaluated the expression of β-globin and γ-globin at the mRNA level using the blood from infant monkey (at birth), 6-month old founder and WT monkeys. And we found that the *HBB* mRNA of the founder at 6 months of age was nearly completely depleted compared to the WT monkey of the same age (>99.9%) (Fig. 1I). Interestingly, the *HBG* mRNA showed a significant increase compared to in the WT adult monkeys and the infant monkey, suggesting that HbF hemoglobin was upregulated in the *HBB* KO founder monkey (Fig. 1I). Then, we quantified the protein levels of HbA1, HbA2 and HbF hemoglobin in this founder. The blood samples of an adult human, an infant human, an infant monkey and four adult WT monkeys were used as controls. Capillary electrophoresis revealed that there were two types of hemoglobin (HbA1 and HbF) in infant human and infant monkey (Fig. 1J). The 6-month-old WT monkey only expressed HbA1, while the *HBB* KO founder only expressed HbF (Fig. 1J). This data demonstrated that the protein of HbA was absent in the *HBB* KO adult founder monkey. Persistence of HbF (named as hereditary persistence of fetal hemoglobin, HPFH) is known to ameliorate β-thalassemia manifestations. Although the HbF persisted in the 6-month old founder monkey, it still showed obvious β-thalassemia symptoms (Fig. 1C–H). This might be caused by unbalance of globin chain, due to different level of α-globin and γ-globin.

Our study showed that *HBB* deficiency resulted in severe β-thalassemia phenotypes in *M*. *fascicularis*. Interestingly, upregulation of HbF was observed in this monkey, which might be caused by genetic compensation reported by two independent groups (El-Brolosy et al., 2019; Ma et al., 2019). Persistence of HbF is known to ameliorate β-thalassemia manifestations, but we still observed serious symptoms in this β-thalassemia monkey. It has been reported that the phenotypes of β-thalassemia patients varied even when co-inherited with HPFH, and some patients still showed serious β-thalassemia symptoms (So et al., 2009). This might be caused by the imbalance between α-globin and γ-globin and the genetic heterogeneity among the populations. Till now, gene editing clinical trials aimed at inducing *HBG* expression to treat β-thalassemia were undergoing. Our data suggested that the level of γ-globin is critical to treat β-thalassemia, so how to regulate its expression precisely by gene editing is an important issue. Collectively, *HBB*-deficient *M*. *fascicularis* represents a valuable model for studying the mechanism of β-thalassemia and long-term safety and efficacy of gene therapy or gene editing therapy targeting *HBB*. Based on our strategy, we can produce more *HBB*-deficient F0 monkeys by using these three gRNAs for further researches. On the other hand, recent progress in monkey somatic cell nuclear transfer (SCNT) also makes it possible to generate more monkeys with the same genetic background (Liu, 2018).

## Electronic supplementary material

Below is the link to the electronic supplementary material.

Supplementary material 1 (PPT 6600 kb)
