# Supplementary material for: HBB-deficient Macaca fascicularis monkey presents with human β-thalassemia
Source: Protein Cell. 2019 May 20;10(7):538–42. doi: 10.1007/s13238-019-0627-y (PMC6588645; doi:10.1007/s13238-019-0627-y)
Supplement: Supplementary file 1 — Supplementary material 1 (PPT 6600 kb) [file 13238_2019_627_MOESM1_ESM.ppt]

## Slide 1
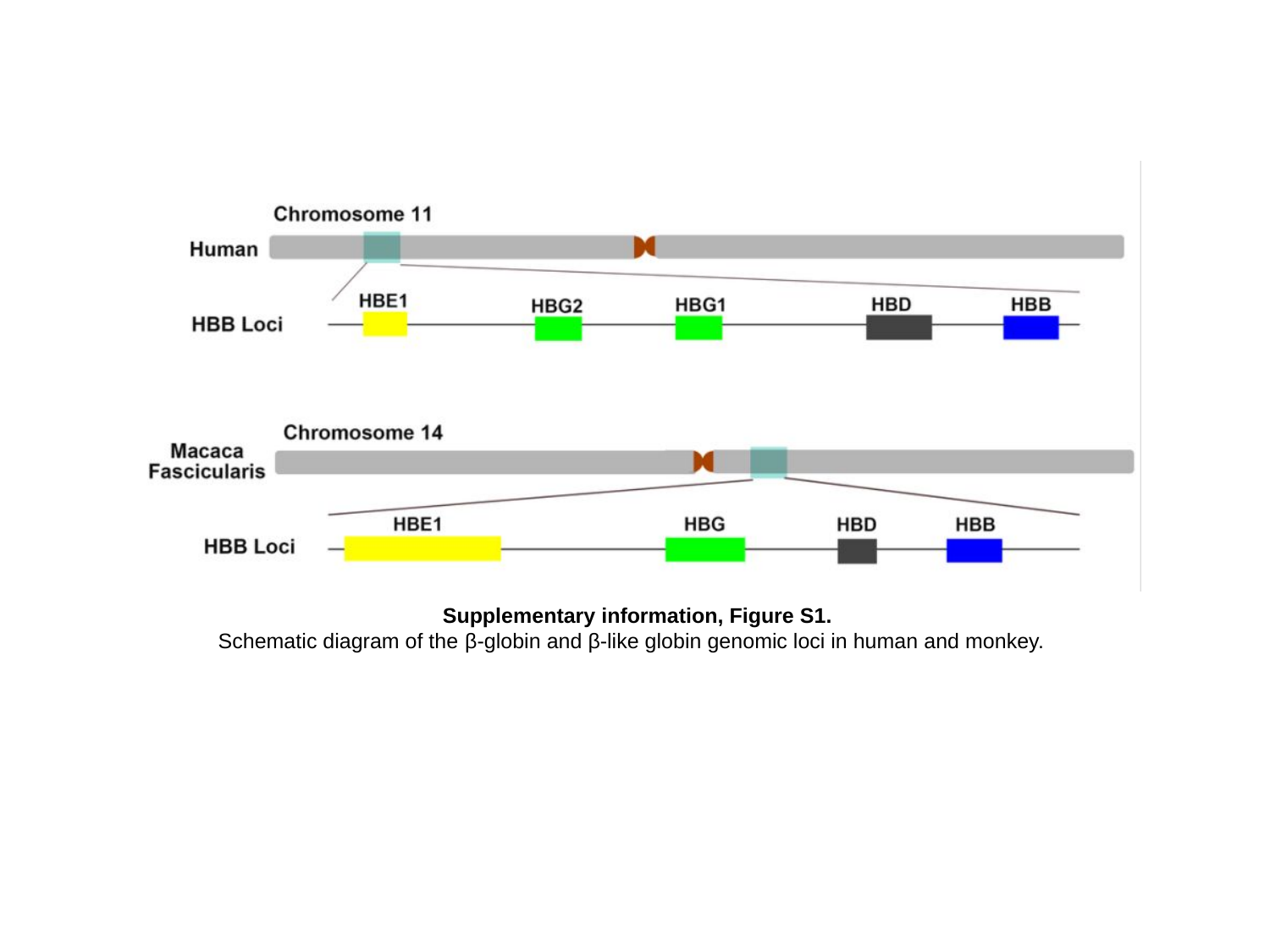

Supplementary information, Figure S1.
Schematic diagram of the β-globin and β-like globin genomic loci in human and monkey.

## Slide 2
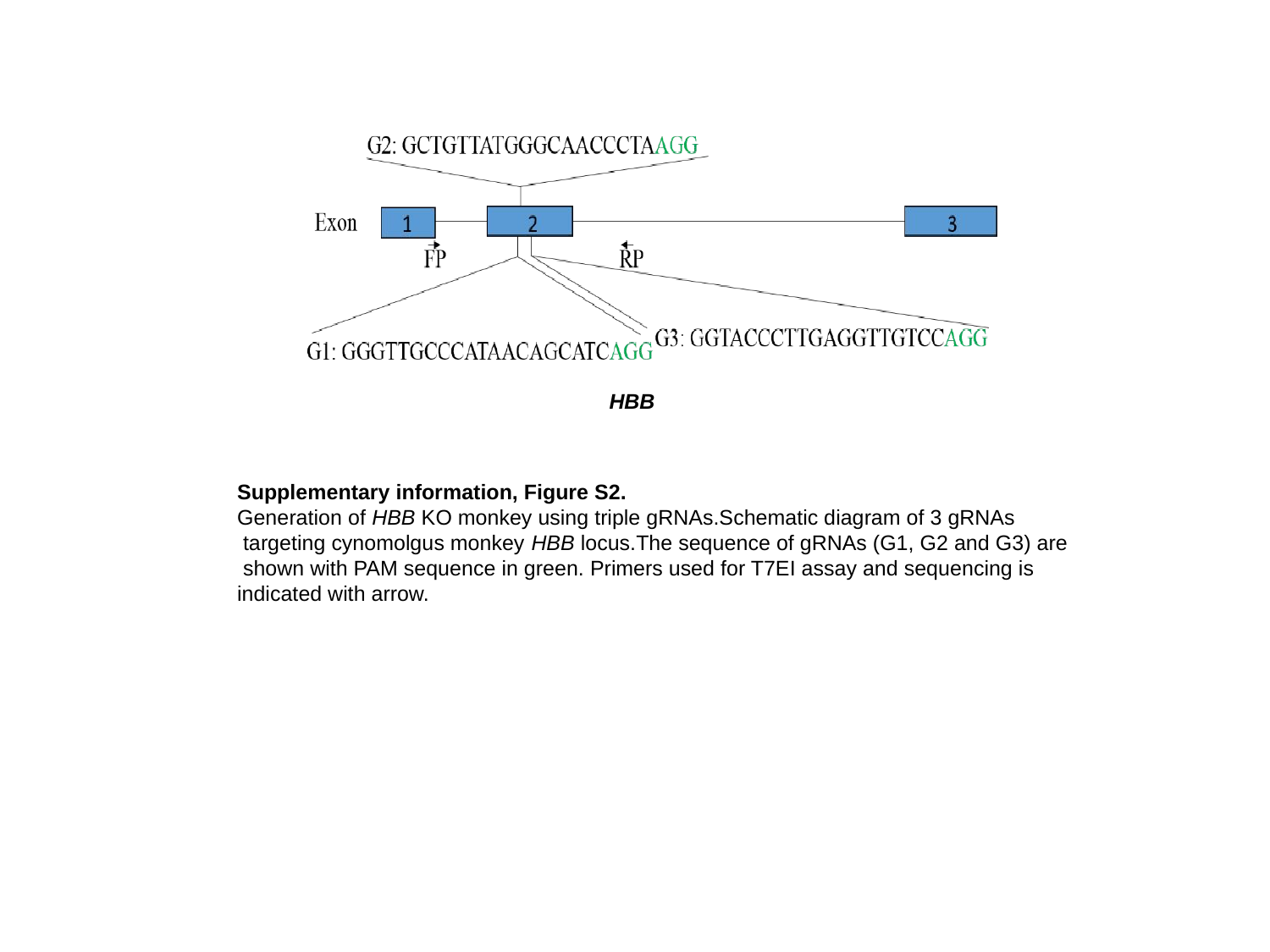

HBB
Supplementary information, Figure S2.
Generation of HBB KO monkey using triple gRNAs.Schematic diagram of 3 gRNAs
 targeting cynomolgus monkey HBB locus.The sequence of gRNAs (G1, G2 and G3) are
 shown with PAM sequence in green. Primers used for T7EI assay and sequencing is
indicated with arrow.

## Slide 3
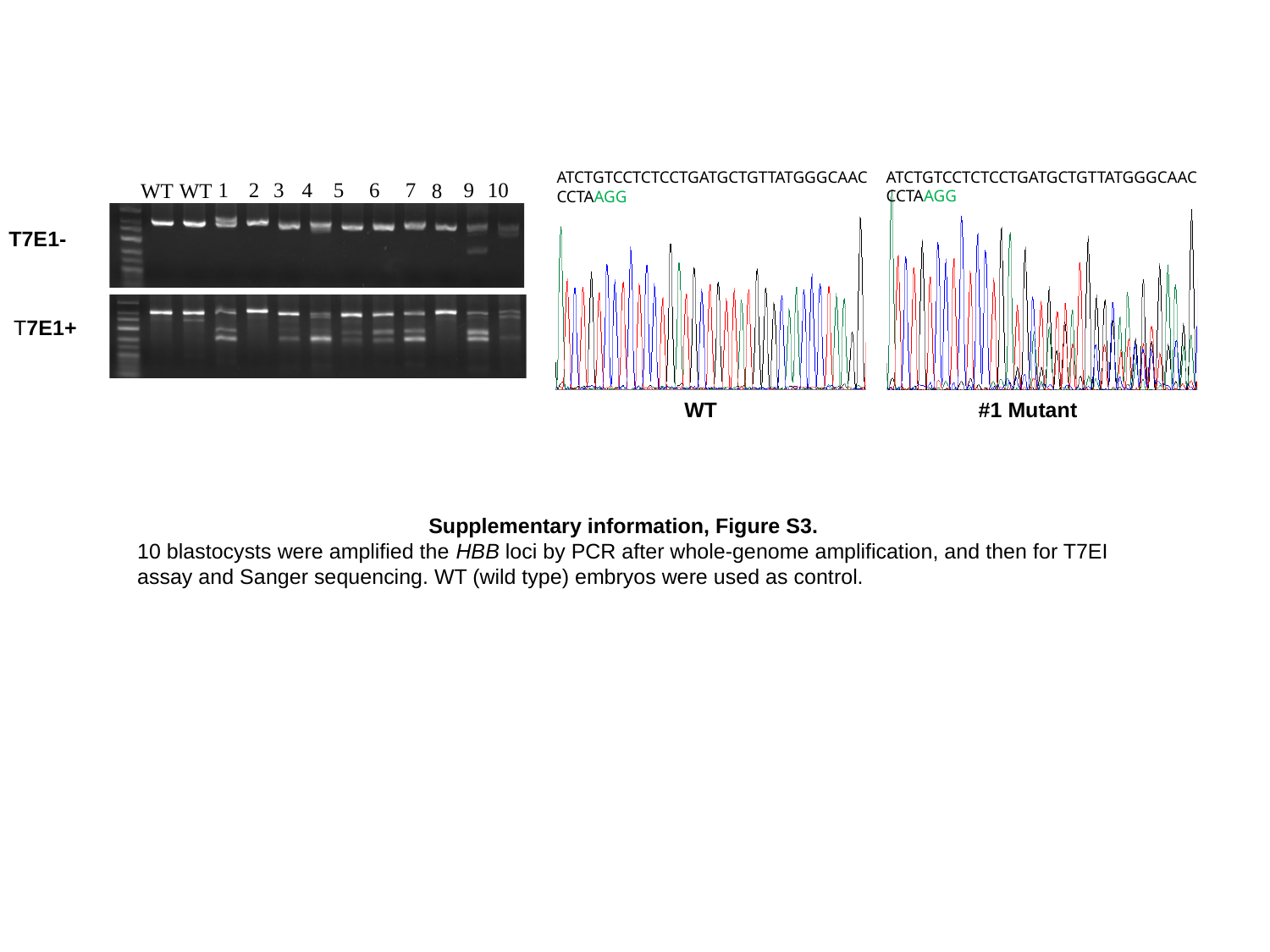

ATCTGTCCTCTCCTGATGCTGTTATGGGCAACCCTAAGG
WT
ATCTGTCCTCTCCTGATGCTGTTATGGGCAACCCTAAGG
#1 Mutant
1
2
3
4
5
6
7
9
10
WT
WT
8
T7E1-
T7E1+
Supplementary information, Figure S3.
10 blastocysts were amplified the HBB loci by PCR after whole-genome amplification, and then for T7EI assay and Sanger sequencing. WT (wild type) embryos were used as control.

## Slide 4
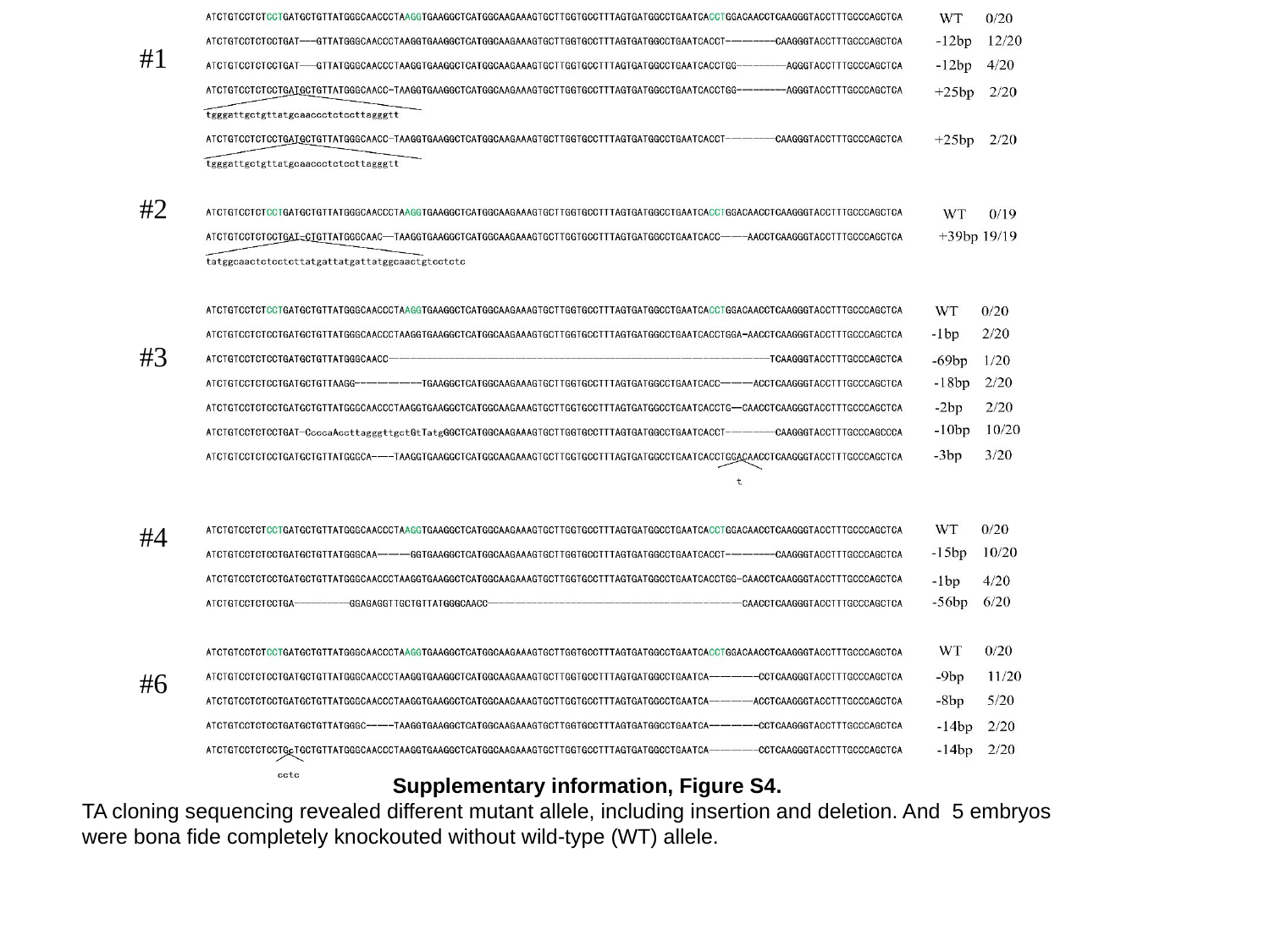

#1
#2
#3
#4
#6
Supplementary information, Figure S4.
TA cloning sequencing revealed different mutant allele, including insertion and deletion. And 5 embryos were bona fide completely knockouted without wild-type (WT) allele.

## Slide 5
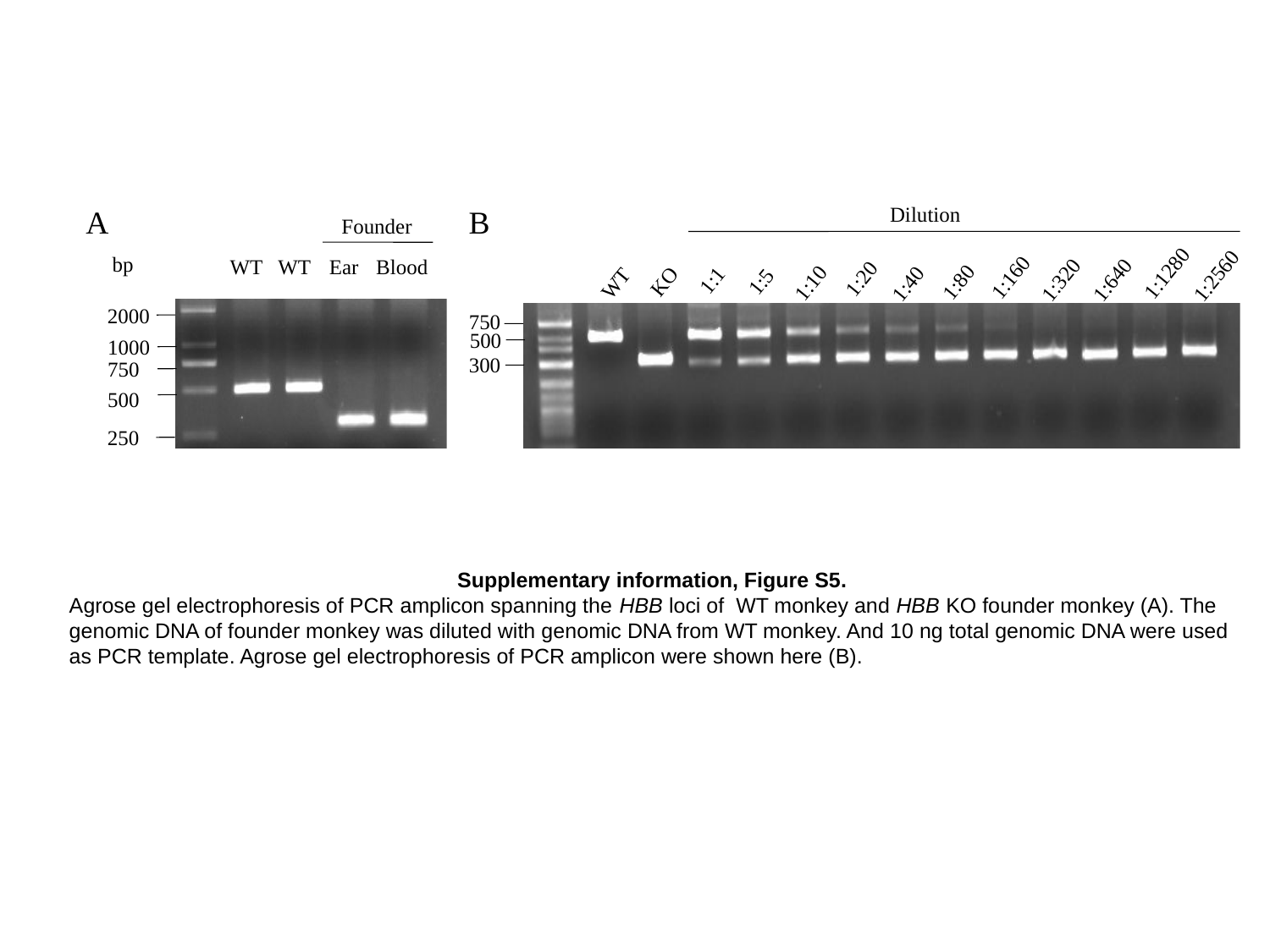

Dilution
1:1280
1:2560
1:1
1:5
1:20
1:160
KO
1:10
1:40
1:80
WT
1:320
1:640
750
500
300
B
A
Founder
bp
WT
WT
Ear
Blood
2000
1000
750
500
250
Supplementary information, Figure S5.
Agrose gel electrophoresis of PCR amplicon spanning the HBB loci of WT monkey and HBB KO founder monkey (A). The genomic DNA of founder monkey was diluted with genomic DNA from WT monkey. And 10 ng total genomic DNA were used as PCR template. Agrose gel electrophoresis of PCR amplicon were shown here (B).

## Slide 6
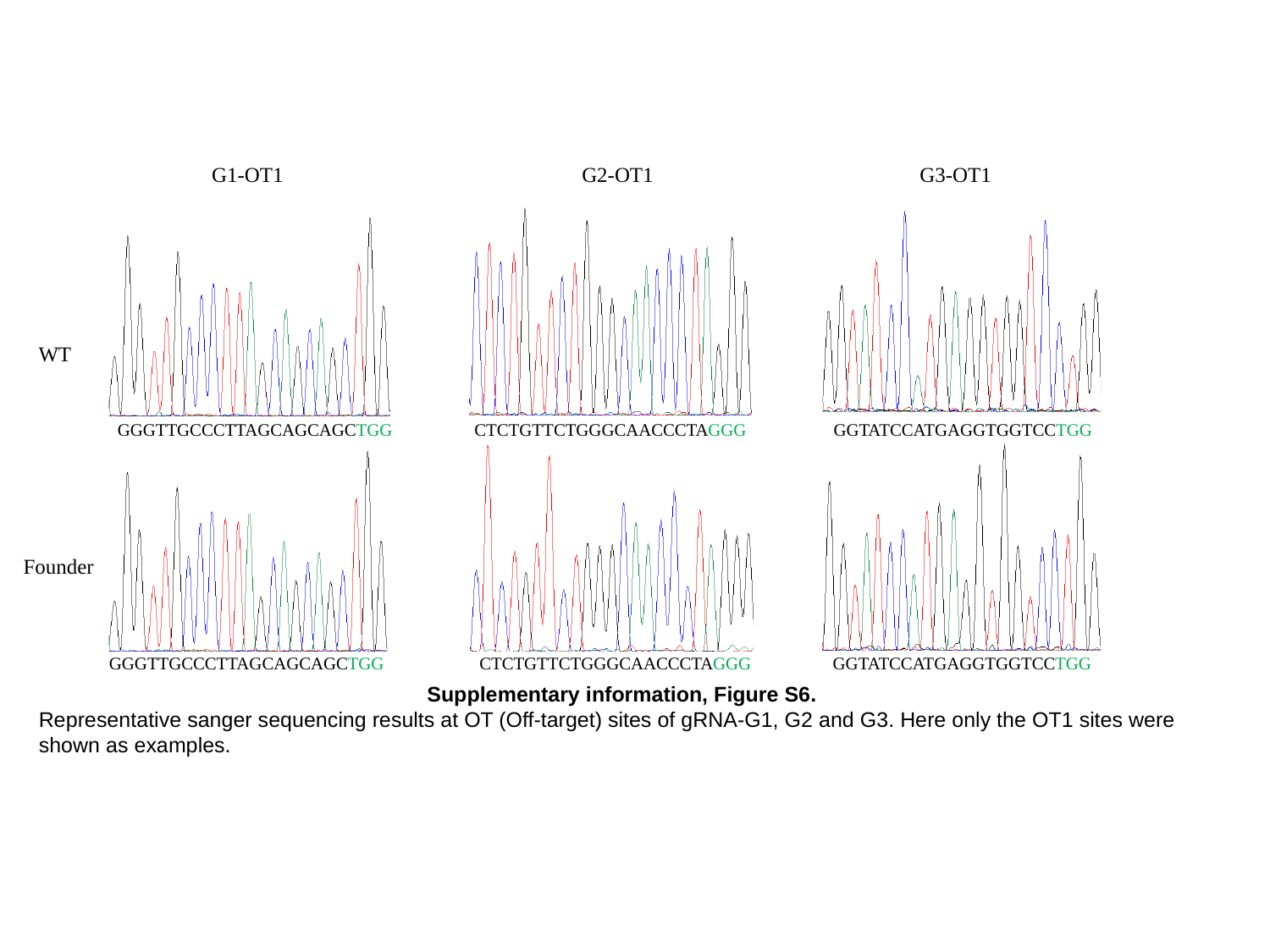

G1-OT1
G2-OT1
G3-OT1
WT
GGGTTGCCCTTAGCAGCAGCTGG
CTCTGTTCTGGGCAACCCTAGGG
GGTATCCATGAGGTGGTCCTGG
Founder
GGGTTGCCCTTAGCAGCAGCTGG
CTCTGTTCTGGGCAACCCTAGGG
GGTATCCATGAGGTGGTCCTGG
Supplementary information, Figure S6.
Representative sanger sequencing results at OT (Off-target) sites of gRNA-G1, G2 and G3. Here only the OT1 sites were shown as examples.

## Slide 7
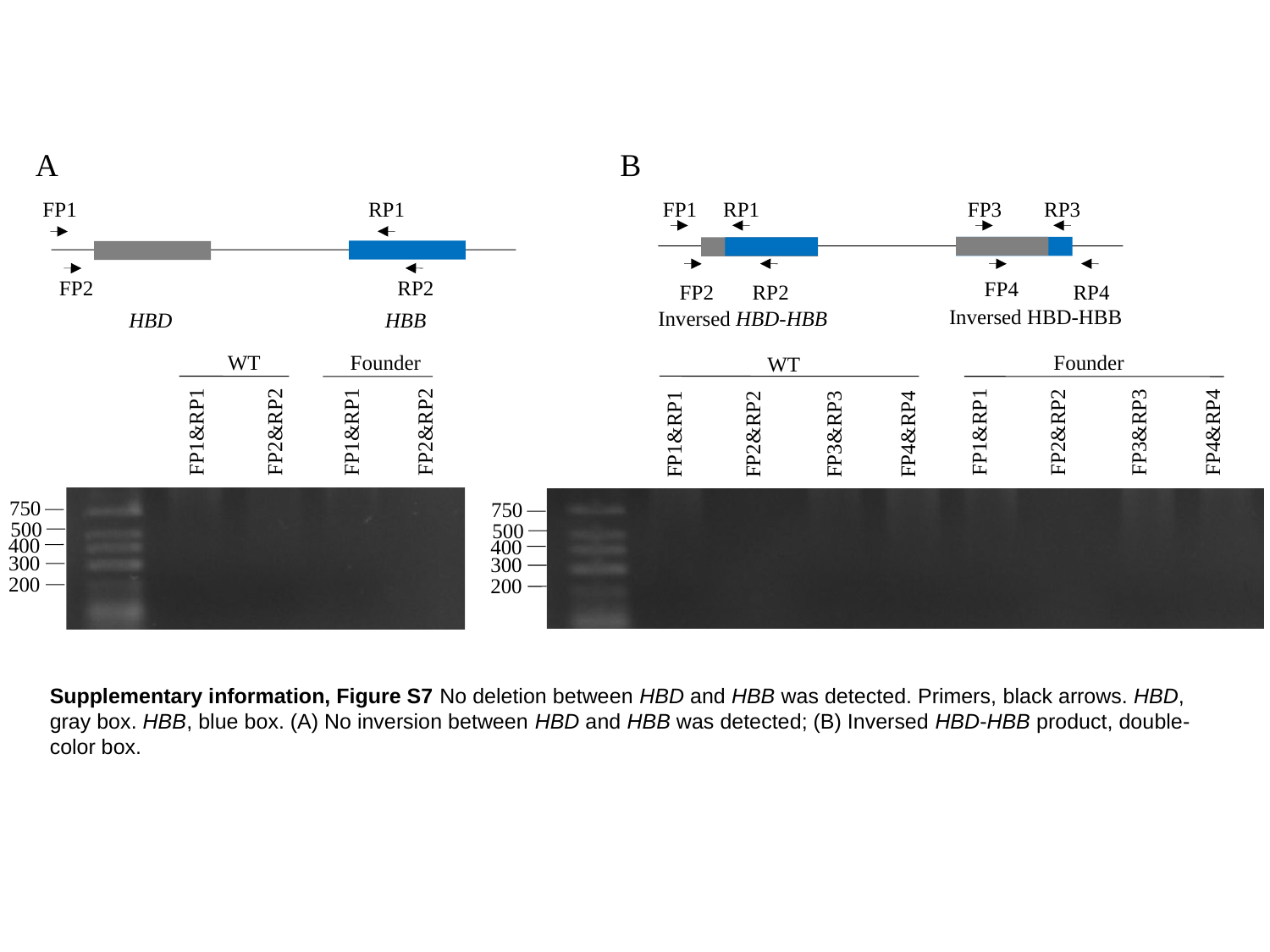

A
B
FP1
RP1
FP2
RP2
HBD
HBB
FP1
RP1
FP3
RP3
FP4
FP2
RP2
RP4
Inversed HBD-HBB
Inversed HBD-HBB
Founder
FP1&RP1
FP2&RP2
FP3&RP3
FP4&RP4
WT
FP1&RP1
FP2&RP2
FP3&RP3
FP4&RP4
750
500
400
300
200
WT
Founder
FP1&RP1
FP2&RP2
FP1&RP1
FP2&RP2
750
500
400
300
200
Supplementary information, Figure S7 No deletion between HBD and HBB was detected. Primers, black arrows. HBD, gray box. HBB, blue box. (A) No inversion between HBD and HBB was detected; (B) Inversed HBD-HBB product, double-color box.

## Slide 8
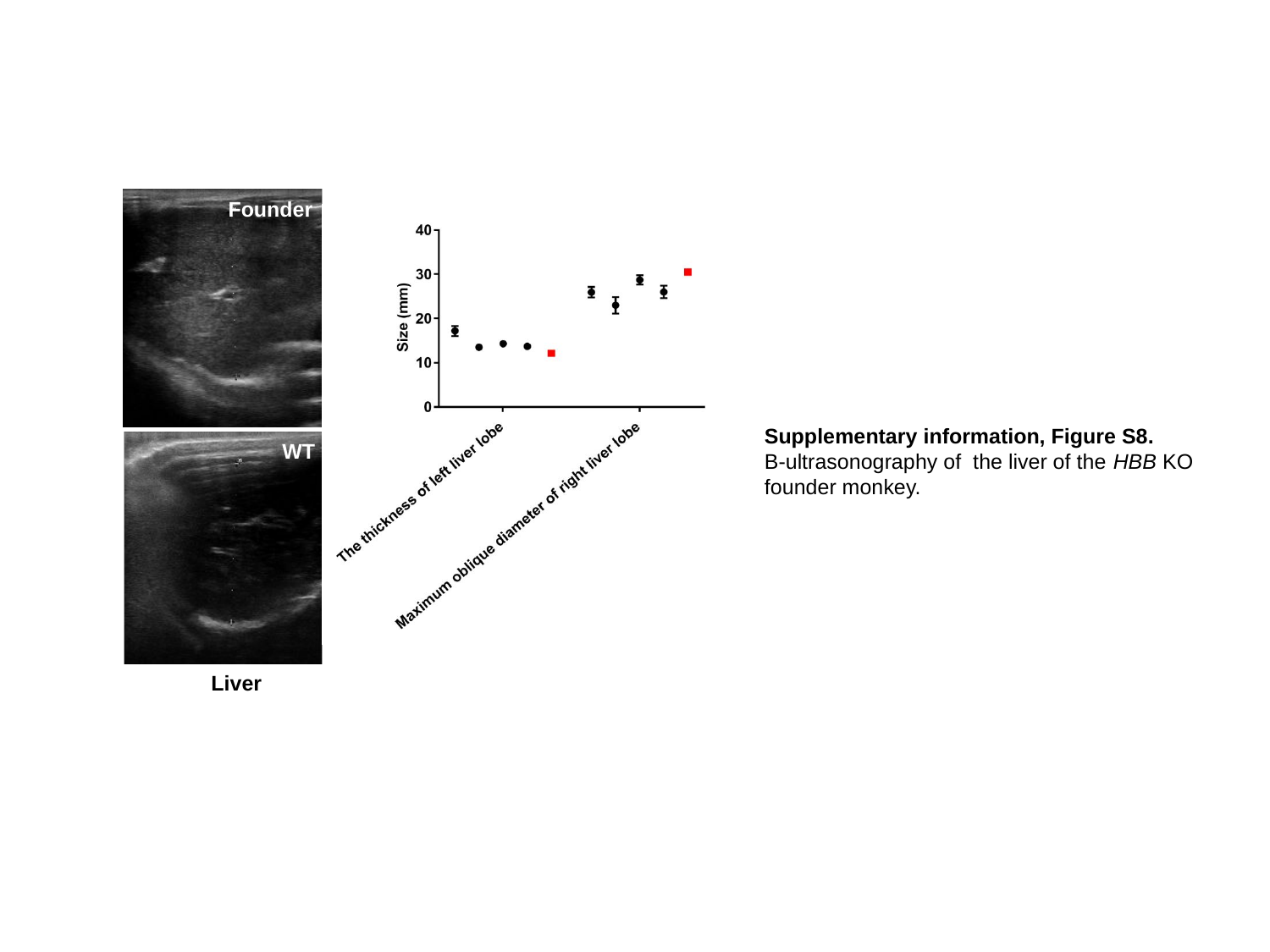

Founder
Supplementary information, Figure S8.
B-ultrasonography of the liver of the HBB KO
founder monkey.
WT
Liver
